# Efficacy of Low-Dose Radioiodine Ablation in Low- and Intermediate-Risk Differentiated Thyroid Cancer: A Retrospective Comparative Analysis

**DOI:** 10.3390/jcm9020581

**Published:** 2020-02-21

**Authors:** Ana María Gómez-Pérez, Jorge García-Alemán, María Molina-Vega, Arantzazu Sebastián Ochoa, Pilar Pérez García, Isabel Mancha Doblas, Francisco J Tinahones

**Affiliations:** 1 Department of Endocrinology and Nutrition, Virgen de la Victoria University Hospital, 29010 Málaga, Spain; jgarcialeman@hotmail.com (J.G.-A.); molinavegamaria@gmail.com (M.M.-V.); aranzatzazuso@hotmail.com (A.S.O.); mandoisa@gmail.com (I.M.D.); fjtinahones@hotmail.com (F.J.T.); 2 Instituto de Investigación Biomédica de Málaga (IBIMA), Virgen de la Victoria University Hospital, 29010 Málaga, Spain; 3 Faculty of Medicine, University of Málaga, 29010 Málaga, Spain; pilarpg93@gmail.com

**Keywords:** differentiated thyroid cancer, radioiodine, low-dose ablation, low-risk, antithyroglobulin antibodies

## Abstract

(1) Background—low-dose radioiodine ablation is an accepted strategy for the treatment of low- and intermediate-risk thyroid carcinomas, although there is no international consensus. The aim of this study is to describe the clinical experience with low-dose radioiodine ablation in patients with low- and intermediate-risk thyroid cancer compared to high-dose ablation. (2) Methods—174 patients with low- and intermediate-risk thyroid cancer, 90 treated with low-dose ablation and 84 treated with high-dose ablation, were included. The primary endpoint was response to treatment one year after ablation, defined by stimulated thyroglobulin, whole body scan and ultrasound imaging. (3) Results—an excellent response rate of 79.8% in the low-dose group and 85.7% in the high-dose group was observed (*p* = 0.049). Stimulated thyroglobulin at the moment of ablation (*p* = 0.032) and positive antithyroglobulin antibodies (*p* < 0.001) were independent predictive factors for nonexcellent response. Young age (*p* = 0.023), intermediate initial recurrence risk (*p* < 0.001) and low-dose ablation (*p* = 0.004) were independent predictive factors for recurrence. (4) Conclusion—low-dose ablation seemed to be less effective than high-dose ablation, especially in those patients with positive antithyroglobulin antibodies or higher stimulated thyroglobulin levels at the moment of ablation. Low dose was associated with higher recurrence rates, and lower age and intermediate initial recurrence risk were independent risk factors for recurrence in our sample.

## 1. Introduction

Differentiated thyroid cancer is the most frequent endocrine neoplasm, with a rising incidence in the last decades in Europe and the United States. This increasing incidence is mainly attributed to the increased diagnosis of clinically nonrelevant papillary microcarcinomas [1,2]. The most frequent histology in differentiated thyroid cancer is papillary thyroid carcinoma (PTC) (90%), followed by follicular thyroid carcinoma (FTC) and less commonly Hürthle cell thyroid cancer (HTC). In most cases the prognosis is excellent, with low recurrences rates and very high survival rates. Finally, we can include poorly differentiated thyroid cancer and anaplastic carcinoma, with a worse prognosis but also with a very low incidence [3,4]. 

Classic thyroid cancer management includes surgery followed by radioiodine ablation. However, in the last years this classic approach has been widely discussed and reviewed to avoid overtreatment of low-risk carcinomas and improve the identification of those tumours with a more aggressive behaviour. In fact, in its last edition, the *American Thyroid Association* (ATA) guidelines for the management of thyroid cancer included the recommendation for an individualized treatment regarding surgery, but also radioiodine (RAI) ablation. After surgery and depending on the initial recurrence risk, the patient could undergo a RAI ablation with a low or high dose [5,6]. 

RAI ablation has a different purpose depending on the initial recurrence risk. In a patient with a low-risk tumour, RAI ablation could be considered in aggressive histologies and a low-dose is an accepted approach to eliminate residual thyroid tissue and facilitate subsequent monitoring. In patients with intermediate-risk tumours, RAI treatment has an adjuvant objective to prevent recurrences and the dose should be individualized (30–50 mCi). Finally, in high-risk tumours, RAI ablation has a therapeutic purpose to eradicate disease, and high doses are preferred [5]. Nevertheless, controversy regarding RAI doses is not solved yet. Two clinical trials have been performed to compare low-dose and high-dose RAI ablation in patients with thyroid cancer. ESTIMABL1 concluded that disease recurrence was not related to RAI ablation strategy in low-risk carcinomas, while [7] in the HiLo study they found noninferior results when comparing low-risk and intermediate-risk tumour recurrence rates with low (<100 mCi) and high doses (≥100 mCi) of RAI [8].

Despite the results of ESTIMABL1 and HiLo suggesting that low-dose RAI ablation is effective in patients with low- and intermediate-risk thyroid cancer, and many subsequent papers supporting this idea [9,10,11], there is no international consensus on RAI dose. In fact, the European Association of Nuclear Medicine and the Society of Nuclear Medicine and Molecular Imaging refused to endorse the ATA 2015 guidelines. To address some of the controversies in this field, those societies published recently the Martinique principles. Among these nine principles, we can highlight three of them related to RAI dose—the need to define RAI therapy as remnant ablation, adjuvant therapy or treatment; optimization of patient selection for RAI therapy according to postoperative disease status; and the optimal dose of RAI cannot be definitively determined from the published literature and needs to be based on multidisciplinary recommendations [12]. 

Therefore, the activity of RAI administered in differentiated thyroid cancer is not clearly defined, and should be based on individualized and multidisciplinary management. The aim of this study was to assess the efficacy of low-dose RAI ablation in patients with low- an intermediate-risk thyroid cancer and compare the results with patients treated with high-dose ablation in the past. 

## 2. Experimental Section

### 2.1. Patients

Since 2014, low-dose RAI ablation has been progressively applied in the Thyroid Cancer office of the Department of Endocrinology at Hospital Universitario Virgen de la Victoria (Málaga, Spain). During this time, more than 90 patients with low- and intermediate-risk thyroid cancer have undergone ablation with low doses of RAI (<100 mCi). Data from a group of patients treated with high doses (≥100 mCi) before 2014 and who, following current guidelines, would be treated with low doses of RAI, were collected. The analysis of the data was performed in 2019. Patients with less than one year of follow-up after RAI ablation were excluded from the analysis. 

To estimate the number of patients that would be necessary to recruit for the retrospective comparative study, we considered a difference of 15% in recurrence rate among subjects treated with low-dose and high-dose ablation as clinically significant. With an alpha error of 0.05, a power of 80% and a loss rate of 15% (loss of samples or other reasons), it would be necessary to include at least 79 subjects in each group. Therefore, we proposed to include at least 80 patients in each group.

### 2.2. Study Protocol

All patients were operated by total thyroidectomy in one or two stages and subsequently referred to the thyroid cancer office. They were postoperatively classified according to the ATA initial recurrence risk classification before they were referred to RAI ablation and this risk state was confirmed with the results of the whole-body scan performed with the treatment. Low-risk category includes differentiated thyroid carcinomas, mostly papillary thyroid or minimally invasive follicular thyroid cancer, limited to the thyroid, with no vascular invasion and no more than five lymph node micrometastases (recurrence risk = 2–5%). Intermediate-risk category encompasses more aggressive histologies, minor extrathyroid or vascular invasion and more than five lymph node metastases less than 3 cm (recurrence risk = 10–30%) [5]. All of them were evaluated one year after the RAI ablation to address the response to treatment according to the dynamic risk stratification scale proposed by Tuttle and adapted by the ATA guidelines [4,5]. Depending on the results of stimulated thyroglobulin (sTg) and antithyroglobulin antibodies (anti-TgAb) measured in the third day of stimulation (with rhTSH or thyroid hormone withdrawal) and the results of whole body scan and ultrasound, patients were classified into four groups—excellent response (sTg < 1 ng/dL, anti-TgAb < 30 UI and negative image), incomplete biochemical response (sTg > 10 ng/dL or rising thyroglobulin and anti-TgAb levels with negative image), incomplete structural response (distant metastases or local disease seen in image studies) and indeterminate response (sTg 1–10 ng/mL or stable or decreasing anti-TgAb levels with unspecific images). For practical reasons due to the small sample size in some groups, the sample was divided into an excellent response and nonexcellent response (including indeterminate, incomplete biochemical and incomplete structural response). Based on previous publications, we defined recurrence as the appearance of new biochemical or structural data of disease and persistence when there was no complete response after the initial treatment [13]. 

### 2.3. Ethics

The study was approved by the Ethical Committee for research of Málaga and was conducted according to the principles of the Declaration of Helsinki. Signed consent has been obtained from each patient after full explanation of the purpose and nature of all procedures used. 

### 2.4. Statistical Analysis

Normal distribution of the variables was evaluated using the Kolmogorov–Smirnov test; variables with Gaussian distribution were expressed as mean (SD). No normal distributed data were expressed as median (25th–75th percentile). For statistical analysis, values of variables that did not have a Gaussian distribution were logarithmically transformed. The hypothesis testing for continuous variables was performed using the ANOVA test (or the Kruskall–Wallis test if non-normality after log transformation). Associations between the qualitative characteristics were tested using the χ2 test. Comparisons were performed for the total sample and also separately for those subjects with low-risk thyroid cancer and intermediate-risk thyroid cancer. The relationship between continuous variables was examined using correlation analyses (Pearson or Spearman). Finally, multivariate logistic regression analysis was performed to estimate odds ratios (ORs) for presenting a nonexcellent response and recurrence, respectively, using the complete sample and also including only the group of patients with low-risk tumours and only the group of patients with intermediate-risk tumours. The variables included in the multivariate regression models were those statistically significant in univariate analyses. 

Significance for all statistical tests was set at *p* < 0.05 for bilateral contrast. All statistical analyses were performed with SPSS version 22.0 for Windows (SPSS Ibérica, IBM, Madrid, Spain). 

## 3. Results

### 3.1. General Characteristics

A total of 174 patients were included in the final analysis, 90 patients treated with low doses and 84 patients treated with high doses. Clinical characteristics and laboratory parameters of the study groups (low RAI dose vs. high RAI dose) are shown in Table 1. There were no significant differences in mean age at diagnosis or sex distribution among the groups. There were some differences in the tumour histology, with a higher percentage of patients with Hürthle cell carcinoma in patients treated with high doses and a higher proportion of mixed tumours in the low-dose group. We also found significant differences in lymph node involvement, with a greater proportion of N1a and N1b in patients treated with high doses of ablation so, consequently, there were differences in this risk level between the groups, with 40.5% of intermediate-risk patients in the high-dose group versus 14.4% of intermediate-risk patients in low-dose group (*p* < 0.001). Anti-TgAb status at the moment of RAI ablation was significantly different between the comparison groups—38.8% of positive anti-TgAb in the low-dose group versus 15.5% in the high-dose group. Finally, regarding qualitative variables, there were significant differences in response to treatment, with a greater percentage of indeterminate responses in the low-dose group and more incomplete biochemical and excellent responses in the high-dose group (*p* = 0.049). Regarding quantitative variables, there were significant differences only in sTg and anti-TgAb one year after the RAI ablation, with higher levels of sTg in the high-dose group (*p* < 0.001) and higher levels of anti-TgAb in the low-dose group (*p* = 0.004). Recurrence rate was somehow higher in low-dose group though there was no statistical significance. 

When we excluded from the analysis patients with intermediate-risk tumours (Table 1), differences in the response to treatment disappeared, maintaining differences in histology, sTg and anti-TgAb levels one year after the treatment. In addition, the marginal effect in the high-dose group was more frequent than in low-dose group when we included only patients with low-risk tumours but there were no differences in recurrence rate. When we compared groups including only patients with intermediate-risk tumours (Table 1), differences in histology among high-dose and low-dose groups disappeared and differences in sTg and anti-TgAb one year after the treatment remained. We found similar differences in response to treatment to those described in the complete sample analysis. However, we found new differences in sex, with a higher proportion of men in the low-dose group, and in tumour size, with more T3 tumours in the low-dose group. Finally, we found significant differences in recurrence rate that was nearly five times more frequent in the low-dose group (*p* = 0.028). 

### 3.2. Association between Clinical Characteristics and Response to Treatment and Recurrence Rate and Predictive Factors for Bad Response and Recurrence

In the correlation analysis there was a negative correlation between age at diagnosis and sTg at the moment of RAI ablation (rho = −0.200, *p* = 0.009) and also with sTg one year after RAI treatment (rho = −0.234, *p* = 0.002). The dose of RAI showed a positive correlation with sTg one year after ablation (rho = 0.532, *p* < 0.001) and a negative correlation with anti-TgAb one year after initial treatment (rho = −0.220, *p* = 0.004). 

Finally, in the multivariate logistic regression analysis, the sTg at RAI ablation showed a direct relation with response to treatment—those patients with higher levels had a greater risk for a nonexcellent response to treatment. Lastly, patients with positive anti-TgAb at the moment of ablation showed a 5.78-fold increased risk for a nonexcellent response compared to those with negative anti-TgAb from the beginning (Table 2). We also performed a multivariate logistic regression analysis to assess the odds of recurrence and we observed an inverse relation between age and recurrence risk. Patients with an initial intermediate risk showed a 15.45-fold increased risk for recurrence, and patients treated with low-dose ablation presented a 12.67-fold increased risk for recurrence (Table 3). 

When we excluded from the analysis patients with intermediate-risk tumours, the results of the multivariate analysis for nonexcellent response were very similar to the results with the whole sample—patients with higher levels of sTg at RAI ablation had a greater risk for nonexcellent response and patients with positive anti-TgAb showed a 8.850-fold increased risk for nonexcellent response (Table 4). However, in the multivariate model for recurrence, we did not find any predictive variable when we included in the analysis only patients with low-risk tumours, with no influence of RAI dose in this kind of patient (Table 5). 

Finally, when we excluded from the analysis patients with low-risk tumours, sTg levels at the moment of ablation still appeared as a predictor of nonexcellent response, but in this risk level positive anti-TgAb did not show any influence on response to treatment. However, patients treated with a low-dose ablation showed a 6.029-fold increased risk for nonexcellent response (Table 4). In the case of recurrence risk, in intermediate-risk tumours the low-dose ablation was the only predictive factor (Table 5). 

Only twelve patients showed recurrence, and therefore we reviewed the characteristics of these patients, finding that lymph node involvement was more frequent in patients with recurrent disease (58.3%) than in patients with no recurrence (19.8%), with statistical significance (*p* = 0.006). Recurrence was also more frequent in patients with intermediate-risk tumours (66.7%) than in patients with low-risk thyroid carcinomas (24.1%), with statistical significance (*p* = 0.003). There were also statistically significant differences in anti-TgAb levels, with higher levels in patients with recurrent disease (*p* < 0.001). No differences were found in other baseline characteristics. 

## 4. Discussion

We have investigated the efficacy of low-dose RAI ablation in our patients with low-risk and intermediate-risk thyroid cancer. Our main results suggest that, though low-dose ablation is an effective strategy in patients with low-risk carcinomas where no RAI therapy is also an acceptable approach, it might be less effective in patients with intermediate-risk carcinomas. In fact, initial recurrence risk was one of the factors positively associated with recurrence rate, and positive anti-TgAb and sTg levels at ablation were the factors positively associated with a nonexcellent response. Low doses of RAI were only associated with recurrence and nonexcellent responses in those patients with intermediate-risk tumours. 

Ablation with low doses of RAI in patients with low- and intermediate-risk thyroid carcinomas is an accepted approach, since the 2015 ATA guidelines included this recommendation [5]. However, there are many controversies around this topic, as evidenced by the fact that the European Association of Nuclear Medicine and the Society of Nuclear Medicine and Molecular Imaging refused to endorse the ATA 2015 guidelines. Even after the meeting of some of these societies in Martinique, the conclusion was that there is no evidence to recommend a dose for RAI ablation and that the decision should be made in a multidisciplinary approach [12]. 

There have been two big clinical trials performed to compare low and high doses of RAI. In ESTIMABL1, disease recurrence was not related to RAI ablation strategy in low-risk carcinomas, with 92% rate of excellent response after low-dose ablation and 98% rate of disease-free survival at 5 years [7]. In the HiLo study, they compared low-risk and intermediate-risk tumour recurrence rates with low (<100 mCi) and high doses (≥100 mCi) of RAI, showing a noninferior result (1–2% risk of recurrence at 5 years in both groups) [8]. Other authors, such as Ghachem [9], Schlumberger [14], Qi [15] or Jiménez-Londoño [11], have reported similar results in observational studies, concluding that low doses were noninferior to high doses in patients with low-risk thyroid cancer. In our study, we observed a higher rate of indeterminate responses in the group treated with low doses of RAI and a higher rate of excellent and incomplete biochemical responses in the group of high-dose ablation, with statistical significance. These differences could be related to the significantly higher frequency of positive anti-TgAb at the moment of ablation in the low-dose group, since anti-TgAb is used to define an indeterminate or incomplete biochemical response [5,6]. The main difference of our study to HiLo was anti-TgAb consideration that was not described in this clinical trial, and with ESTIMABL1 the difference was the inclusion of intermediate-risk tumours. Nevertheless, the rate of excellent response in the low-dose group was 78.9%, and this result is comparable to previous studies [8,9,11,14,15]. 

We also found a higher rate of recurrence in the low-dose group, but in this case the differences did not reach statistical significance. The inclusion of intermediate-risk patients could be related to the results we have found. In fact, we found that those significant differences found in response to treatment were only observed in the intermediate-risk group, and that recurrence rate differences reached statistical significance in this group. In this point, it is important to comment that differences in sex and tumour size in the intermediate-risk group could also have an influence on poorer outcomes in patients treated with low doses. 

In studies of correlation we found some interesting data, such as the positive correlation of RAI dose and sTg one year after ablation and the negative correlation between RAI dose and anti-TgAb one year after the treatment. The negative correlation between age and sTg at the moment of ablation is also remarkable, suggesting a worse postsurgical state in younger patients. Based on these correlations, we looked for predictive factors of a nonexcellent response and recurrence. We found that sTg at the moment of ablation was an independent risk factor for nonexcellent response in the complete sample, but also when we separated patients with low- an intermediate-risk tumours. These results are similar to the results reported by Ghachem et al [9], who found that patients with Tg after surgery <10 ng/mL were more likely to have a complete response, and Fallahi et al, who found that a Tg<4.5 ng/mL was predictive of a complete response [16]. Tg levels after surgery and in RAI ablation have been studied as factors affecting the response to treatment and recurrence rates by many authors [17,18,19,20], and our results seemed to be in the same direction. Although postsurgical sTg is not standardized, it could be interesting clinical information to take into account. However, the factor more strongly associated with a higher risk of nonexcellent response was positive anti-TgAb at the moment of ablation, mainly due to indeterminate response. Many studies have addressed the importance of the evolution of anti-TgAb in predicting recurrence or persistent disease in patients with thyroid cancer [21,22,23]. In fact, the 2015 ATA guidelines included anti-TgAb titre evolution as a factor of response [5]. In this point, it is important to take into account that there is a prevalence of 10% of positive anti-TgAb in the general population and it rises to 25% in patients with thyroid cancer [24]. 

Regarding the risk of recurrence, we found that a higher age was a protective factor, and that this differs from the data reported for example by Verburg et al [25], who found a higher specific mortality for thyroid cancer and recurrence rate in patients > 45 years treated with low-dose compared to those treated with high-dose ablation. But this effect of age in our sample could be due to a confounding factor, as it disappeared when we separated the analysis by recurrence risk. Finally, we found that initial recurrence risk and a low-dose ablation were independent predictors for recurrence in our sample. These results are in contrast with those reported by HiLo, where recurrence rates were not associated with RAI ablation strategy [7,8], and also with the results of comparative analyses published to date [9,10]. In this point, it is also interesting to remark that when we took into account only patients with low-risk thyroid cancer, the RAI dose had no relation to recurrence risk, but when we analyzed only patients with intermediate-risk tumours, RAI dose was a strong predictor of recurrence, suggesting that low-dose ablation might be a better strategy in patients with low-risk thyroid cancer than in patients with intermediate-risk tumours. 

Our study had some important limitations. First, it was an observational study performed comparing a sample of selected patients with low- and intermediate-risk thyroid cancer to be treated with a low-dose ablation with a historic cohort of patients treated with high-dose. There were some statistically significant differences among those groups. Differences in histology could be related to the recent changes in histologic classification of thyroid tumours [3]. There were also differences in lymph node involvement and initial risk of recurrence, although the patients included in the analysis would have been treated with low doses following our current strategy. Changes in the classification of patients and follow-up also could have influenced these differences and thereby the results of our analysis. The follow-up period was quite short in some patients, especially in the low-dose group. Another limitation is that we had no data about BRAF status in our sample, a factor that could influence prognosis and outcomes of the disease. 

## 5. Conclusions

In conclusion, low-dose ablation might be an effective approach for the treatment of low-risk and intermediate-risk thyroid cancer, but in our sample it seemed to be less effective than high-dose ablation, especially in those patients with positive anti-TgAb or higher sTg levels at the moment of ablation. Low doses were associated with higher recurrence rates in patients with intermediate-risk tumours, and in fact, initial recurrence risk was an independent risk factor for recurrence in our sample. However, more data from prospective studies are needed to solve the remaining doubts regarding RAI dose in thyroid cancer treatment, and it would be interesting to compare low-dose with no RAI therapy in low-risk carcinomas. 

## Figures and Tables

**Table 1:** Baseline clinical characteristics. Comparison between groups—low-dose vs. high dose ablation.

	Complete Sample		Low-Risk Tumours		Intermediate-Risk Tumours	
	Low-Dose	High-Dose	Low-Dose	High-Dose	Low-Dose	High-Dose
*N*	90	84	77	50	13	34
Age at diagnosis, mean (±SD) (years)	45.7 (13.9)	43.3 (14.08)	46.04 (14.32)	44.98 (13.62)	43.69 (±11.34)	40.82 (±14.58)
Sex (%)					*	*
Male	16.7	13.1	13	14	38.5	11.8
Female	83.3	86.9	87	86	61.5	88.2
Histology (%)	*	*	*	*		
PTC	46.7	58.3	46.8	60	42.6	55.9
FTC	13.3	7.1	14.3	4	7.7	11.8
Mixed	8.9	0	9.1	0	7.7	0
HTC	1.1	7.1	0	0	7.7	17.6
PTC + HC	4.4	0	2.6	0	15.4	0
FVPTC	25.6	27.4	27.3	36	15.4	14.7
Multifocality (%)						
No	72.2	63.1	72.7	56	69.2	73.5
Yes	27.8	36.9	27.3	44	30.8	26.5
Margins (%)			*	*		
Free	70	63.1	71.4	70	61.5	52.9
Near	11.1	15.5	9.1	20	23.1	8.8
Contact capsule	5.6	15.5	6.5	10	0	23.5
Capsule invasion	13.3	6	13	0	15.4	14.7
Tumour size (%)					*	*
T1	63.3	67.9	62.3	76	69.2	55.9
T2	33.3	32.1	36.4	24	15.4	44.1
T3	3.3	0	1.3	0	15.4	0
Lymph node affection (%)	*	*				
N0	85.6	69	95.3	100	38.5	23.5
N1a	13.3	27.4	6.5	0	53.8	67.6
N1b	1.1	3.6	0	0	7.7	8.8
Initial recurrence risk (%)	**	**	–	–	–	–
Low	85.6	59.5				
Intermediate	14.4	40.5				
Anti-TgAb-RAI ablation (%)	*	*	*	*	*	*
Negative	61.2	84.5	62.5	80	53.8	91.2
Positive	38.8	15.5	37.5	20	42.6	8.8
Response to treatment (%)	*	*			*	*
Excellent	78.9	85.7	84.4	86	42.6	85.3
Indeterminate	14.4	3.6	11.7	6	30.8	0
IBR	2.2	6	1.3	6	7.7	5.9
ISR	4.4	4.8	2.6	2	15.4	8.8
Recurrence rate (%)					*	*
No	90	96.4	94.8	100	61.5	91.2
Yes	10	3.6	5.2	0	38.5	8.8
sTg at RAI ablation, median (ICR) (ng/dL)	0.87 (0.12–2.47)	1.15 (0.20–3.15)	0.84 (0.11–2.58)	0.50 (0.20–2.23)	1.30 (0.15–2.85)	1.61 (0.30–5.43)
Anti-TgAb-RAI ablation, median (ICR) (UI)	20 (15–33.28)	20 (16.05–20.58)	20 (15–30.50)	20 (20–23.75)	17 (15–177.50)	20 (15–20)
sTg 1 year, median (ICR) (ng/dL)	0.05 (0.04–0.20)	0.20 (0.20–0.20)	0.04 (0.04–0.20)	0.20 (0.20–0.20)	0.05 (0.04–0.20)	0.20 (0.20–0.47)
Anti-TgAb 1 year, median (ICR) (UI)	15 (9.61–20)	8.41 (4.71–15)	15 (8.65–18)	8.41 (4.80–15)	17 (12.27–47.45)	8.40 (4.55–17.67)

SD, standard deviation; PTC, papillary thyroid cancer; FTC, follicular thyroid cancer; HTC, Hürthle cell thyroid cancer; PTC + HC, papillary thyroid cancer with Hürthle cells; FVPTC, follicular variant of papillary thyroid cancer; RAI, radioiodine; anti-TgAb, antithyroglobulin antibodies; sTg, stimulated thyroglobulin; ICR, interquartile range. * *p* < 0.05. ** *p* < 0.001.

**Table 2:** Bivariate and Multivariate Logistic Regression Analysis—odds of nonexcellent response. Complete sample.

	Bivariate			Multivariate		
Independent variables	OR	95% CI	*p*	OR	95% CI	*p*
Age	0.973	0.944–1.002	0.07	0.971	0.938–1.004	0.087
Lymph node involvement						
N0	1 (ref.)			1 (ref.)		
N1	2.398	1.026–5.609	**0.044**	1.113	0.267–4.641	0.883
Initial recurrence risk						
Low	1 (ref.)			1 (ref.)		
Intermediate	0.482	0.211–1.098	0.082	0.407	0.147–1.123	0.082
RAI dose (mCi)	0.996	0.985–1.007	0.43	0.994	0.981–1.007	0.361
RAI dose						
Low	1 (ref.)			1 (ref.)		
High	0.667	0.300–1.484	0.321	1.062	0.510–22.294	0.969
Anti-TgAb						
Negative	1 (ref.)			1 (ref.)		
Positive	0.202	0.088–0.462	**<0.001**	5.785	2.431–13.762	**<0.001**
sTg at the moment of RAI	1.032	0.997–1.069	0.073	1.043	1.004–1.084	**0.032**
Anti-TgAb at the moment of RAI	1.004	1.000–1.007	**0.027**	1.002	0.999–1.005	0.285
sTg at the moment of RAI						
<1 ng/dL	1 (ref.)					
1–10 ng/dL	1.194	0.520–2.743	0.676	–	–	–
>10 ng/dL	1.543	0.376–6.329	0.547			

RAI, radioiodine; sTg, stimulated thyroglobulin; anti-TgAb, antithyroglobulin antibodies. (–) These variables were analysed in bivariate logistic regression but they were excluded in the multivariate analysis because they were not statistically significant. Significant results are highlighted in bold.

**Table 3:** Bivariate and Multivariate Logistic Regression Analysis—odds of recurrence. Complete sample.

	Bivariate			Multivariate		
Independent variables	OR	95% CI	*p*	OR	95% CI	*p*
Age	0.926	0.869–0.987	**0.018**	0.933	0.879–0.991	**0.023**
Lymph node involvement						
N0	1 (ref).			1 (ref.)		
N1	5.687	1.694–19.093	**0.005**	2.536	0.404–15.921	0.321
Initial recurrence risk						
Low	1 (ref.)			1 (ref.)		
Intermediate	0.159	0.045–0.555	**0.004**	15.456	3.369–70.907	**<0.001**
RAI dose						
Low	12.596	1.848–85.841	**0.01**	12.679	2.280–70.516	**0.004**
High	1 (ref.)			1 (ref.)		
Anti-TgAb						
Negative	1 (ref.)			1 (ref.)		
Positive	2.952	0.892–9.587	0.076	2.915	0.703–12.088	0.141

RAI, radioiodine; anti-TgAb, antithyroglobulin antibodies. Significant results are highlighted in bold.

**Table 4:** Multivariate Logistic Regression Analysis—odds of nonexcellent response (separate analysis based on initial risk of recurrence).

	Only Low-Risk			Only Intermediate-Risk		
Independent variables	OR	95% CI	*p*	OR	95% CI	*p*
Age	0.966	0.920–1.015	0.168	–	–	–
RAI dose						
Low	–	–	–	6.029	1.152–31.545	**0.033**
High				1 (ref.)		
Anti-TgAb						
Negative	1 (ref.)			–	–	–
Positive	8.850	2.644–29.626	**<0.001**			
sTg at the moment of RAI	1.145	1.014–1.294	**0.029**	1.054	1.002–1.109	**0.042**
Anti-TgAb at the moment of RAI	1.011	1.000–1.023	0.054	–	–	–

RAI, radioiodine; sTg, stimulated thyroglobulin; anti-TgAb, antithyroglobulin antibodies. (–) These variables were analysed in bivariate logistic regression but they were excluded in the multivariate analysis because they were not statistically significant. Significant results are highlighted in bold.

**Table 5:** Multivariate Logistic Regression Analysis—odds of recurrence (separate analysis based on initial risk of recurrence).

	Only Low-Risk			Only Intermediate-Risk		
Independent variables	OR	95% CI	*p*	OR	95% CI	*p*
Age	0.940	0.853–1.035	0.208	0.925	0.854–1.002	0.055
Lymph node involvement						
N0	1 (ref)			–	–	–
N1	6.191	0.467–82.010	0.167			
RAI dose	–	–	–			
Low				11.728		
High				1 (ref.)	1.655–83.081	**0.014**
Anti-TgAb						
Negative	1 (ref.)			1 (ref.)		
Positive	7.412	0.745–73.776	0.088	0.845	0.0.079–8.999	0.889

RAI, radioiodine; anti-TgAb, antithyroglobulin antibodies. (–) These variables were analysed in bivariate logistic regression but they were excluded in the multivariate analysis because they were not statistically significant. Significant results are highlighted in bold.
